# Molecular Aspects of Dopaminergic Neurodegeneration: Gene-Environment Interaction in Parkin Dysfunction

**DOI:** 10.3390/ijerph8124702

**Published:** 2011-12-16

**Authors:** Syed F. Ali, Zbigniew K. Binienda, Syed Z. Imam

**Affiliations:** Neurochemistry Laboratory, Division of Neurotoxicology, National Center for Toxicological Research, US Food and Drug Administration, Jefferson, AR 72029, USA; Email: zbigniew.binienda@fda.hhs.gov (Z.B.); syed.imam@fda.hhs.gov (S.Z.I.)

**Keywords:** Parkinson’s disease, parkin, gene, environment, dopaminergic

## Abstract

Parkinson’s disease (PD) is a common neurodegenerative movement disorder that is characterized pathologically by a progressive loss of midbrain dopaminergic neurons and by protein inclusions, designated Lewy bodies and Lewy neurites. PD is one of the most common neurodegenerative diseases, affecting almost 1% of the population over 60 years old. Although the symptoms and neuropathology of PD have been well characterized, the underlying mechanisms and causes of the disease are still not clear. Genetic mutations can provide important clues to disease mechanism, but most PD cases are sporadic rather than familial; environmental factors have long been suspected to contribute to the disease. Although more than 90% of PD cases occur sporadically and are thought to be due, in part, to oxidative stress and mitochondrial dysfunction, the study of genetic mutations has provided great insight into the molecular mechanisms of PD. Furthermore, rotenone, a widely used pesticide, and paraquat and maneb cause a syndrome in rats and mice that mimics, both behaviorally and neurologically, the symptoms of PD. In the current review, we will discuss various aspects of gene-environment interaction that lead to progressive dopaminergic neurodegenration, mainly focusing on our current finding based on stress-mediated parkin dysfunction.

## 1. Introduction

Parkinson’s disease, initially described as “shaking palsy” in the early 1800s by British physician James Parkinson, is among the most prevalent neurological disorders, particularly in the population over 60 years of age. The current figure of at least four million affected individuals worldwide is predicted to double by the year 2040, as the elderly population increases. The most prominent clinical features are bradykinesia, rigidity, resting tremor, and postural instability, with some patients also experiencing cognitive, autonomic, and psychiatric manifestations. Pathologically, PD is a relentlessly progressive neurodegenerative disease that is characterized by the loss of dopaminergic neurons in the substantia nigra pars compacta [1]. Protein aggregation, a feature common to many neurodegenerative disorders, results in the formation of Lewy bodies, a neuropathological hallmark of PD [2,3]. Various provocative evidence suggest that environmental exposures to certain neurotoxicants (heavy metals, pesticides and fungicides) may play a role in the development of neurodegenerative movement disorders such as Parkinson’s disease (PD). A number of large association studies have identified factors that may correlate with altered risk for developing PD, and these studies have shown both genetic and environmental factors playing a role in this risk [4,5,6,7]. However, the concept that gene-environment interactions may play a role in PD pathogenesis, have been addressed by very few studies able directly in an experimental system. 

## 2. Gene-Environment Interplay

**Figure 1:**
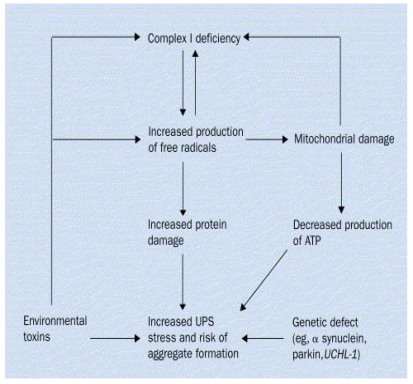
An interplay between gene and environment affecting various stages of progressive mechanisms leading to the neuronal death in PD. The central generation of free radicals after exposure to environmental toxins or decreased UPS function and protein aggregate formation due to genetic defect is thought to be a major mechanism of dopaminergic cell death in PD.

Various studies suggest that PD develops from complex gene-environment interactions which involve a crosstalk among multiple molecular pathways leading to PD neurodegeneration. The following diagram illustrates interplay between gene and environment affecting various stages of progressive mechanisms leading to the neuronal death in PD (Figure 1).

The findings illustrating the role of causative gene mutations in PD have led to numerous mechanisms leading to neuronal degeneration in PD. Major categories of these mechanisms can be categorized into protein aggregation such as Lewy Body and neuritis, impaired function of ubiquitin-proteasome system (UPS) leading to impaired protein degradation and accumulation of toxic proteins, mitochondrial dysfunction, specially Complex I deficiency, and finally oxidative stress leading to various signal transductions disruptions. Following Table [8] lists various genes whose mutation is attributed to development of familial PD (Table 1).

**Table 1:** Loci and genes associated with familial PD or implicated in PD [8].

Locus	Chromosome Location	Gene	Inheritance Pattern	Typical Phenotype
PARK1& PARK4	4q21-q23	*α-synuclein*	AD	Earlier onset, features of DLB common
PARK2	6q25.2-q27	*parkin*	Usually AR	Early onset with slow progression
PARK3	2p13	*unknown*	AD, IP	Classic PD, sometimes dementia
PARK5	4p14	*UCH-L1*	unlcear	Classic PD
PARK6	1p35-p36	*PINK1*	AR	Early onset with slow progression
PARK7	1p36	*DJ-1*	AR	Early onset with slow progression
PARK8	12p11.2-q13.1	*LRRK2*	AD	Classic PD
PARK10	1p32	*unknown*	unclear	Classic PD
PARK11	2q36-q37	*unknown*	unclear	Classic PD
N/A	5q23.1-q23.3	*Synphilin-1*	unclear	Classic PD
N/A	2q22-q23	*NR4A2*	unclear	Classic PD

N/A—not assigned, AD—Autosomal Dominant, AR—Autosomal Recessive, IP—Incomplete Penetrance, DLB—Dementia with Lewy Bodies.

The α-synuclein (*SNCA*) mutations and single-nucleotide polymorphisms (SNPs) make α-synuclein adopt a propensity for misfolding and accelerated aggregate formation. Excessive α-synuclein aggregates may overwhelm UPS protein degradation. Accumulated α-synuclein can translocate to the mitochondria and impair mitochondrial activity. *Parkin* mutations and *UCHL-1* SNPs prevent the proteolytic degradation of excessive toxic proteins (e.g., misfolded α-synuclein) in proteasomal machinery.

**Figure 2:**
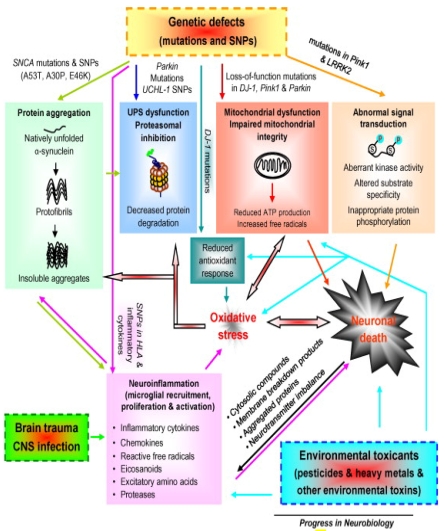
Gene-environment interplay induces complex crosstalk leading to the increased level of oxidative stress in dopaminergic neurons. Various pathways and their dysfunctions resulting from genetic defects in PD-related genes such as α-synuclein, Parkin, DJ-1, LRRK2, PINK 1 lead to molecular mechanisms that result in an increased oxidative stress. Similarly, stress and trauma that might be physical or induced by environmental toxicants can inhibit proper functions of the gene products of PD-related genes such as Parkin, DJ-1 or PINK 1 or induce mitochondrial Complex I inhibition or neuroinflammation resulting in increased oxidative stress thus leading to neuronal cell death [9].

*PINK1*, *Parkin*, and *DJ-1* functionally interact to maintain mitochondrial integrity and functionality and to protect cells against adverse effects of multiple stressors. Mutations in these genes cause mitochondrial dysfunction and subsequent decline in ATP production and increase in free radical generation, which results in oxidative stress and energy deficiency. Impaired mitochondria can release cytochrome *c* and other ‘pro-apoptotic factors’ triggering apoptotic cascades and cell death. Mitochondria in at least some forms of PD reveal abnormal morphology, impaired fission-fusion balance, and metabolic malfunction. *DJ-1* mutations reduce antioxidant response of cells, aggravating oxidative stress. Oxidative stress engages in diverse cellular processes and plays a prominent role in the induction of neuronal death. For instance, excessive production of free radicals can damage proteins (e.g., abnormal modification of α-synuclein and inactivation of Parkin), lipids, DNA, or RNA, leading to cell dysfunction (e.g., UPS and mitochondrial impairment) and eventual death. Mutations in *Pink1* and *LRRK2* induced aberrant kinase activity, altered substrate specificity, leading to inappropriate protein phosphorylation (e.g., increased α-synuclein phosphorylation at serine 129 by LRRK2 *in vitro*) and thereby affecting cell survival. Environmental toxins and brain trauma can trigger neuronal lesions by damaging mitochondria, causing oxidative stress, inducing inflammation in the central nervous system (CNS), and compromising defence mechanisms of cells. Some environmental risk factors can directly activate microglia (the resident immune cells in the CNS) or cause systemic inflammation, which in turn affects CNS inflammation. Genetic variation and polymorphisms in the *HLA* region and several inflammatory cytokines may become risk factors for PD. Activated microglia produce and secrete a spectrum of inflammatory and cytotoxic molecules, such as cytokines, chemokines, reactive free radicals, eicosanoids, and proteases. In addition to modulating microglial activity, these molecules influence the fate of surrounding neurons. Excessive inflammatory reaction usually becomes exaggerated and destructive, and turns into chronic inflammation that drives progressive neurodegenerative process. Injured neurons activate the surrounding microglia through the release or leakage of noxious self-compounds into the extracellular milieu, such as membrane breakdown products, abnormally processed or aggregated proteins (e.g., α-synuclein and β-amyloid), imbalanced neurotransmitters (e.g., elevated glutamate) and cytosolic compounds (e.g., α-synuclein, ATP, HMGB1 and neuromelanin). Thus, gene–environment interplay induces complex crosstalk among multiple signal cascades, forming a network and culminating in neuronal death and PD development (Figure 2) (paragraph and diagram adapted from Gao and Hong [9]).

## 3. Parkin Modification as an Example of Gene-Environment Interaction in PD

Much current evidence suggests that impaired regulation of protein aggregation and dysfunction of the ubiquitin-proteasome system (UPS) is a common pathway in the progression of both genetic and sporadic forms of PD [4,5,6]. The UPS mediates the ubiquitination of a substrate by a multi-step enzymatic process, which includes a ubiquitin activator (E1), a ubiquitin conjugator (E2), and a ubiquitin ligase (E3). Ubiquitinated substrates are then targeted for degradation by the proteasome [10]. Parkin, one of a number of E3 protein-ubiquitin ligases [11], mediates ubiquitination of itself, as well as an unusually large number of other protein substrates, including the α-SYN-interacting protein synphilin-1 (and other synaptic proteins), PaelR (parkin-associated endothelin-like receptor), cyclin E, α/β tubulin, and the p38 subunit (p38/JTV-1) of the aminoacyl-tRNA synthetase complex [12], which has recently received the alternative designation of aminoacyl-tRNA Synthetase (ARS)-interacting multifunctional protein type 2 (AIMP2) [13]. The gene that encodes parkin was originally demonstrated to have an association with autosomal recessive juvenile-onset parkinsonism in Japanese families [14]. Current evidence suggests that up to half of hereditary parkinsonism and 10% of all early-onset PD cases are associated with *PARKIN* mutations [15,16].

Although clinical phenotypes vary, patients with *PARKIN* mutations generally develop parkinsonism at an early age, exhibit slow disease progression, and respond well to levodopa therapy. A subset of these mutations results in the loss of parkin E3 ubiquitin ligase function , which is thought to lead to UPS dysfunction, aggregation of parkin and/or its ligase substrates, and degeneration of dopaminergic neurons [4,5,17]. Mutations in parkin are currently recognized as one of the most common cause of familial Parkinsonism. To date, the descriptions of parkin-related PD include patients with homozygous and compound heterozygous mutations, as well as those with a single mutated allele [16]. Although PD due to parkin mutations is classically transmitted in an autosomal recessive inheritance, the existence of patients with single mutations raises the possibility of an expanded risk associated with parkin haploin sufficiency [16,17,18]. Supporting this possibility is the recent association of parkin gene promoter polymorphisms with late-onset PD [19]. Parkin variability, both qualitative and quantitative, could thus be considered as a risk factor for the development of PD. 

The importance of parkin expression in neuronal survival is probably related to the multitude of 1neuroprotective roles it appears to serve [20]. Parkin apparently confers protection to neurons against a diversity of cellular insults, including manganese-induced cell death [21], α-synuclein toxicity [22], proteasomal dysfunction [23], endoplasmic reticulum stress [24], Pael-R [25] and AIMP2 (p38/JTV-1) [13] accumulation, and kainate-induced excitotoxicity [26]. Additionally, parkin confers neuronal resistance to stimuli that promote mitochondria-dependent apoptosis and dopamine-mediated toxicity [27,28]. Given the multiplex neuroprotective roles of parkin, it is conceivable that any depletion in the level or activity of parkin would significantly compromise neuronal integrity. Indeed, many familial PD-linked mutations of *PARKIN* cause a loss of parkin catalytic competency [29,30]. Similarly, the inhibition of parkin activity by BAG5 enhances dopamine neuron death in an *in vivo* model of PD [31]. Conversely, animals with over-expressed parkin have reduced α-synuclein-induced neuronal pathology compared with normal control animals [32,33]. Susceptibility of primary neurons of parkin null mice to rotenone has been shown to be significantly high. In addition to the role of parkin in neuronal survival, a very recent study suggested a protective role of parkin against mitochondrial toxins and β-amyloid accumulation in skeletal muscles during Inclusion Body Myositis [34]. 

The *PARKIN* gene, located at the PARK2 locus on chromosome 6q, is comprised of 12 exons encoding a 465 amino acid protein that is expressed widely, but most prominently in muscle and brain [14]. The ~52 KDa parkin protein (see Figure 2) is comprised of an N-terminal ubiquitin-like (UBL) domain, a unique parkin domain (UPD), and two RING (really interesting new gene) fingers flanking an IBR (in-between-ring) domain at the C terminus. All of these domains appear to be important, since PD mutations are found within each of them [10]. Studies by Ted Dawson [35] and Philipp Kahle [36], have shown that post-translational modifications of parkin protein alter its E3-ubiquitin ligase activity. In the first instance, Chung, Dawson, and their colleagues have shown that S-nitrosylation of parkin compromises its ubiquitin ligase activity and abrogates its protective function against α-synuclein-mediated neurotoxicity. This group also demonstrated extensive S-nitrosylation of parkin in mouse models of PD, as well as in PD patients [35]. In the second example, Yamamoto, Kahle, and colleagues [36] have recently shown that parkin phosphorylation on various serine residues results in a decrease in its E3 ubiquitin ligase activity. However, no evidence has yet been obtained for the induction of phosphorylation of parkin by oxidative or nitrative stress or for the presence of phosphorylated parkin in PD. Moreover, a recent study observed vulnerability of parkin to modification by dopamine, the principal transmitter lost in PD, suggesting a possible mechanism for the progressive loss of parkin function in dopaminergic neurons [37]. Taken together; these studies suggest that critical modifications of parkin play an important role in the pathogenesis of sporadic PD, the predominant form of the disease. Since sporadic PD is thought to be due in part to oxidative stress [38,39], the work of Chung and colleagues provides a link between oxidative damage and the role of parkin in sporadic PD. Therefore, oxidative, nitrative, or nitrosative stress, and more recently dopaminergic stress, are thought to impair the function of parkin through post-translational modification and/or altering the solubility of parkin [37,40,41]. The molecular mechanisms underlying the impairment of parkin’s function by these stressors are unknown. Moreover, the extent to which these modifications play a role in the common sporadic form of Parkinson’s disease has not yet been defined. 

An etiologic link has been suggested between PD and the herbicide paraquat (1,1'-dimethyl-4,4'-bipyridinium) [42,43]. Paraquat is structurally similar to MPP+, the active metabolite of MPTP. Epidemiologic data suggest a positive dose-response relationship between lifetime cumulative exposure to paraquat and risk of PD [44]. In experimental studies in which paraquat has been administered to animals, researchers have observed loss of SN dopaminergic neurons, depletion of dopamine in the SN, reduced ambulatory activity, and apoptotic cell death [45].

The insecticide rotenone induces clinical and pathologic features in rats similar to those induced by PD, including selective degeneration of the nigrostriatal dopaminergic system and movement disorders [46]. Synergistic effects have been observed in animals administered a combination of rotenone and lipopolysaccharide, a molecule that stimulates inflammation [47,48]. Susceptibility to rotenone has been shown to be increased in the neurons from parkin null mice [34]. In our recent studies [49], we show that tyrosine phosphorylation of parkin by c-Abl, a tyrosine kinase activated majority by oxidative stress, is a major post-translational modification that leads to loss of parkin function and disease progression in sporadic PD. Moreover, inhibition of c-Abl offers new therapeutic opportunities for blocking PD progression. 

**Figure 3:**
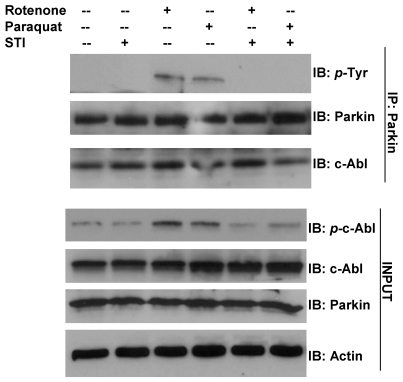
Environmental toxin stress results in tyrosine phosphorylation of parkin in SH-SY5Y cells expressing myc-parkin. Cells were treated with 250 μM paraquat or 2.5 μM rotenone for 24 hr. In some cases, cells were pretreated with 10 μM STI-571 6 hours prior to toxin treatment. RIPA lysates were prepared and subjected to immunoprecipitation with anti-parkin antibody and immunoblotted with antibodies as shown in the figure.

In our studies, c-Abl was activated and parkin was tyrosine phosphorylated in SH-SY5Y cells treated with either paraquat or rotenone (Figure 3). Furthermore, pre-treatment with STI-571 inhibited the paraquat or rotenone mediated activation of c-Abl and tyrosine phosphorylation of parkin. These data provide support for the activation of c-Abl mediated pathway and loss of parkin function during the exposure to environmental toxins such as paraquat and rotenone, leading the progression of PD after these exposures. 

## 4. Conclusions

Recent evidence has demonstrated a close interplay between genetic and environmental causes of PD neurodegeneration. Environmental risk factors and PD-associated gene mutations have been shown to act in parallel pathways, likely sharing some common molecular mechanisms. Previously, we have shown that chronic rotenone administration can lead to significant injury to the nigro-striatal system, mediated by increased generation of nitric oxide [50]. A very recent study presents molecular insights into link between gene products associated with development of PD and the pesticides. Gu and colleagues [51] took some of the first steps toward unraveling the molecular dysfunction that occurs when proteins are exposed to environmental toxins. Their discovery helps further explain the link between PD and two particular pesticides—rotenone and paraquat. This study provides the evidence that oxidative stress, possibly due to sustained exposure to environmental toxins, may serve as a primary cause of PD suggesting why many people, such as farmers exposed to pesticides, have an increased incidence of the disease. Scientists previously believed that PD might be associated with oxidative stress, which is when electronically unstable atoms or molecules damage cells. Gu and colleagues specifically demonstrated how oxidative stress caused parkin proteins to cluster together and malfunction, rather than performing normally by cleaning up damaged proteins. More studies are needed to establish solid relationship between various gene products responsible for the development of PD and the impact of different environmental factors on these gene products during the development and progression of sporadic PD. 
